# Discovery of Deep-Water Bamboo Coral Forest in the South China Sea

**DOI:** 10.1038/s41598-019-51797-3

**Published:** 2019-10-29

**Authors:** Jianru Li, Pinxian Wang

**Affiliations:** State Key Laboratory of Marine Geology, Tongji University, Shanghai, 200092 China

**Keywords:** Biological techniques, Marine biology

## Abstract

A deep-water coral forest, characterized by slender and whip-shaped bamboo corals has been discovered from water depths of 1200–1380 m at the western edge of the Xisha (Paracel Islands) area in the South China Sea. The bamboo corals are often accompanied by cold-water gorgonian “sea fan” corals: *Anthogorgia* sp. and *Calyptrophora* sp., as well as assemblages of sponges, cirrate octopuses, crinoids and other animals. The coral density increased toward the shallower areas from 24.8 to 220 colonies per 100 m^2^ from 1380 m to 1200 m water depth. This is the first set of observations of deep-water bamboo coral forests in Southeast Asia, opening a new frontier for systematic, ecological and conservation studies to understand the deep-coral ecosystem in the region.

## Introduction

Even if known to science for over a century, deep-sea cold-water corals have become a research focus only in the 1990s, when advanced acoustics and submersibles started to reveal the complexity of coral ecosystems in the deep ocean. The first discovery was cold-water, scleractinian coral reefs in the Atlantic Ocean^1,2^, followed by gorgonian coral forests in various parts of the global ocean^3^. Intriguing is the rarity of such reports from the Northwest Pacific. Along with some Russian data from Kamchatka^4^, the only case in the international literature is the report of gorgonian corals in the Shiribeshi Seamount west of Hokkaido, Japan^5^.

**Figure 1:**
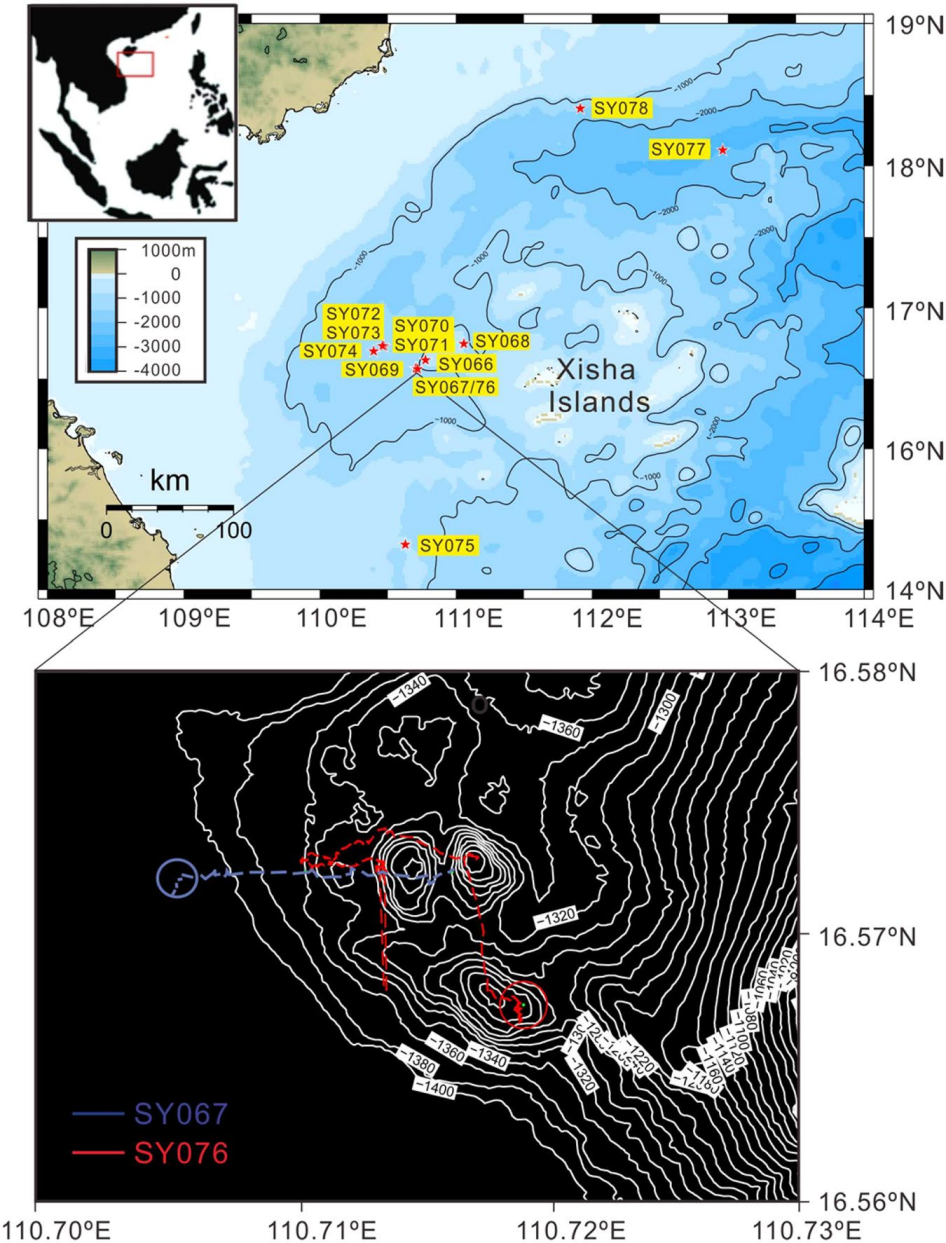
Location of 2018 dive sites in the Xisha Islands area, SCS.

Here we report, for the first time, on a deep-water cold coral forest in the South China Sea (SCS), which was observed between 1200 and 1380 meters depth during an expedition in May 2018, with the manned submersible “*ShenhaiYongshi*” to the Xisha Islands (Paracel Islands) area (Fig. 1, Table 1).

**Table 1:** Brief description of visual sequences where deep-water coral gardens were observed in the NW South China Sea.

Dive	Transect number	Water depth	Length	Bamboo corals		Sea fan corals				total corals	
				*Lepidisis* sp.		*Anthogorgia* sp.		*Calyptrophora* sp.			
		(m)	(m)	n	colonies/100 m^2^	n	colonies/100 m^2^	n	colonies/100 m^2^	n	colonies/100 m^2^
SY067	1	1374.9	28.4	50	29.3					50	29.3
	2	1369.3	32.8	54	27.4	6	3.0			60	30.5
	3	1354.4	13	28	35.9					28	35.9
	4	1343.8	48.4	71	24.4					71	24.4
	5	1332.7	25.4	88	57.7	2	1.3			90	59.1
	6	1283.6	39	67	57.3					67	57.3
	7	1239.2	40	47	39.2	23	19.2	35	29.2	105	87.5
	8	1222	30.4	79	86.6	12	13.2	3	3.3	94	103.1
SY076	9	1212.5	13.8	24	69.6	13	37.7			37	107.2
	10	1201.8	18	74	117.5	10	15.9			84	133.3
	11	1215.5	45	103	76.3	76	56.3	13	9.6	192	142.2
	12	1199.1	43	119	69.2	234	136.0	35	20.3	388	225.6
	13	1347.2	18.4	25	34.0			2	2.7	27	36.7
	14	1331.8	40.4	124	76.7			1	0.6	125	77.4

**Figure 2:**
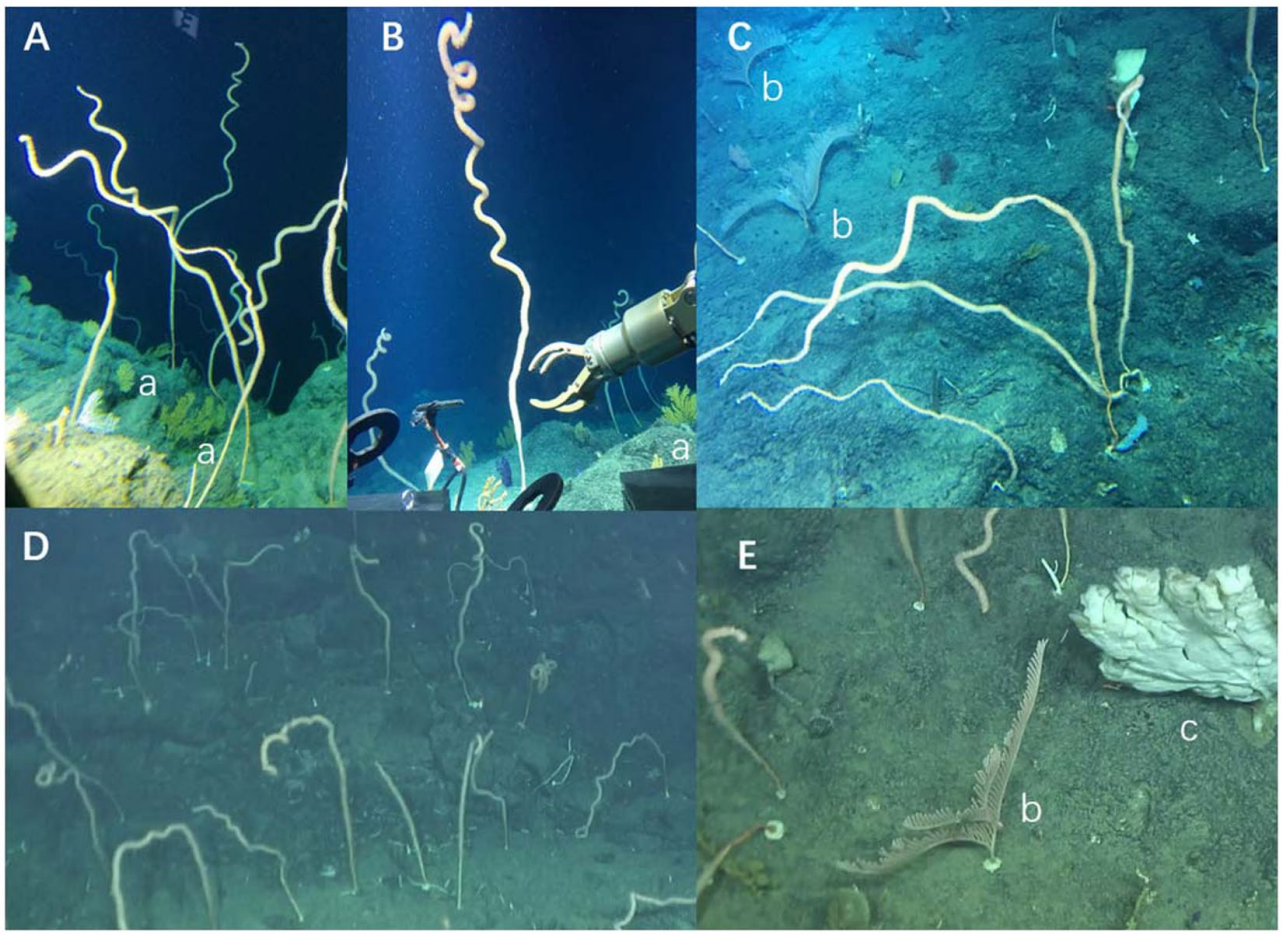
Deep-water bamboo coral forest of *Lepidisis* from the Xisha Islands area, NW South China Sea. (**A**) Bamboo corals with sea fans *Anthogorgia* (a); (**B**) A colony of bamboo coral *Lepidisis* sp. over 2 m in height; (**C**) Bamboo corals with sea fans *Calyptrophora* (b); (**D**) Bamboo coral forest; (**E**) Sea fan *Calyptrophora* (b), sponge (c) and bamboo corals.

The discovery of the coral forest was a surprise, as deep-water corals were known for decades from the SCS in occasional in dredge samples. Most striking were the tall, whip-shaped bamboo corals, standing in patches sometimes densely along the submersible track (Fig. 2). The whip-like coral colonies frequently exceed 2 m in height, but with the curling upper part where polyps grow the entire colony body may stretch up to 5 m in length. Bamboo corals are among the most easily recognized deep-water octocorals due to their articulated skeleton. Although the precise taxonomic identification is still under revision, the Xisha bamboo corals are temporarily assigned to a single species *Lepidisis* sp., because *Lepidisis* is the only genus with whip-shaped unbranched colony of the bamboo coral family Isididae^6,7^, albeit the “branching” based taxonomy remains a matter of debate^7,8^.

As an understory shrub in forest, the bamboo corals are accompanied with other cold-water gorgonian corals, basically two forms of “sea fans”: *Anthogorgia* sp. and *Calyptrophora* sp. (Table 1; Fig. 2). The *Anthogorgia* colony is relatively small and about 20 cm high (“a” in Fig. 2A,B), whereas *Calyptrophora* is much broader and taller, about 40 cm in height (“b” in Fig. 2C,E). Sponge is the most important group of sessile animals in the coral forest (“c” in Fig. 2E), sometimes making up a “sponge ground” of the sea floor. The bamboo coral forest appears to be acting as a habitat provider for many supra-benthos fauna, and associated animals such as cirrate octopuses and crinoids were observed during the cruise.

**Figure 3:**
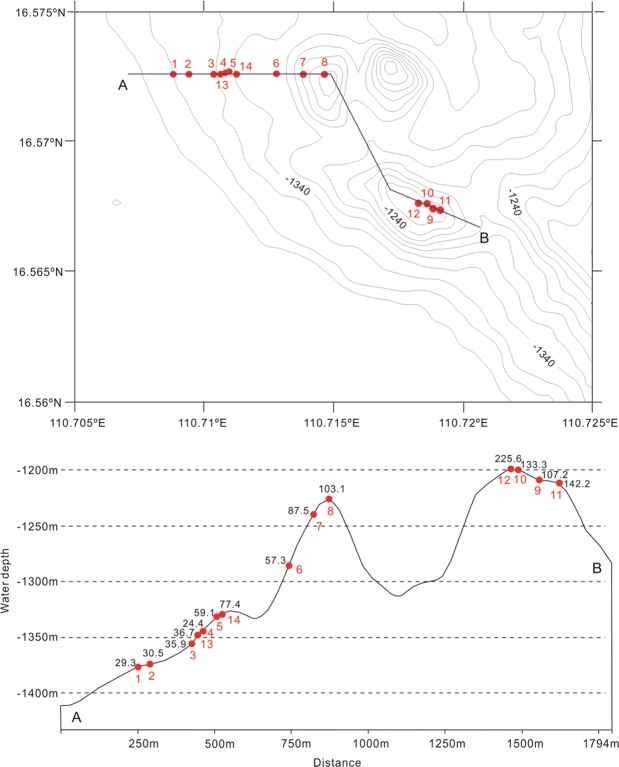
Depth distribution of colony density of deep-water corals along the lower slope of Ganquan reef, South China Sea (see Table 1).

The occurrence of this bamboo-coral forest was beyond our expectation, as the deep-diving cruise with manned submersible was designed to explore drowned coral reefs in the Xisha Islands area. Like in Hawaii^9^, the Xisha carbonate platform features a number of submerged terraces and sunken reef with the reef flat now lying hundreds meters below sea level. The bamboo-coral forest is located at the foot of the submerged Ganquan reef, near the western border of the Xisha Islands (Fig. 1). A total of ten dives (No. SY066-074, 076) covered the Ganquan reef from its top at about 600 m deep to ~1400 m on its western slope. Although scattered gorgonian corals were encountered during most of the dives, the densely populated coral forest was found only during dives No. SY067 and SY076, along the lower slope at the southwestern corner of Ganquan reef. There, the colony density increased upwards from 24.8 to 220 colonies per 100 m^2^ from 1380 m to 1200 m water depths (Fig. 3; Table 1).

Deep coral ecosystems thriving in dark and cold waters, and a host of favorable conditions has to come together to support the growth of “animal forests”, including topographic features, water current, and food supply^10^. In our case, the occurrence of bamboo-coral forest on the Ganquan slope is readily explained by a “hilly” topography, a sunken reef substrate, and active currents of nutrient-rich water. Remarkable was the intense showers of “marine snow”, the organic detritus falling from the upper layers of the water column, driven by active currents in the coral forest^11^. The current was also vividly reflected in the whip-like move of the bamboo corals, as well as the orientation of the sea fans.

Relatively dense gorgonian corals were also observed in a 2000 m deep canyon (dive No. SY077 and SY078) and at the rim of a mud volcano about 600 m deep (dive No. SY075) (see “Methods” below), respectively in the north and south of the Xisha Islands area (Fig. 1), but densely populated forest was so far found only at the Ganquan slope. To the East, deep-water gorgonian forests with bamboo corals were recently found also on seamounts in the deep SCS basin. However, it remains unclear how broadly the deep-water coral forests are distributed there. As the large, suspension feeding colonies of deep-water corals play a major role in pelagic-benthic coupling, which comprises a missing link in biological pump, knowing its distribution in a sea basin like the SCS is crucial.

Among various deep-sea corals, the bamboo coral forest is of particular interest. The bony, hard-bodied calcareous bamboo corals may really resemble bamboo trees on land and probably cover large deep sea areas. Current information indicates that bamboo corals of the family Isididae are nearly cosmopolitan in distribution and may reach 4850 meters depth, and they have been found in the North Atlantic, Japan, Indonesia, the Hawaiian Islands, New Zealand^12,13^, and off Alaska^7^. Adding to this list now is the discovery reported here that deep-water gorgonian forests, including bamboo coral forests, were observed on seamounts and sunken reef flats in the deep SCS basin. As the global diversity centre of tropical reef-building corals^14^, Southeast Asia including the SCS must have been hosting highly diverse deep-coral communities, which will require many more systematic surveys before their ecosystem can be fully understood.

## Methods

In order to obtain the density data of coral colonies, a semi-quantitative analysis was made on the basis of video records from individual dives. The manned submersible “*ShenhaiYongshi”* was equipped with two parallel laser beams, and the laser beams provided a 10 cm scale for measuring the width of the video frame. During the 10 dives (Fig. 1), transects varied between 13 m and 48 m in length, estimated from the navigation data. A total distance of 638 m along the seabed was video-recorded, covering an area of approximately 1856 m^2^. The total time of the video records was 51.7 min. Results of two dives (SY067, SY076) are listed in Table 1 as examples.

Video-type of each dive was split into a number of frame sequences, and abundance of coral colonies was estimated by dividing the number of colonies within a video sequence with the approximate area of the sequence, while the number of coral colonies was counted directly from the videos. Two sources of errors may present in the estimated area of video sequences: inaccurate geographical positions, and variable width of the visual field^10^. Unfortunately, there was no practical way to estimate the width of the visual field continuously, which varied with the height above bottom and the pitch and roll angles of the video-camera lens. However, most of the time the inspection platforms were kept 2.5–3 m above the bottom, with very little variation in the pitch and roll. The variable field width probably induced an error in coral abundance <15%, but the width estimation of the video frame sometimes suffered from the laser beam problem and increased the errors in area estimates. The errors in area estimates has a much smaller influence on the abundance estimates at large scale (whole transects) than at a small scale (video sequences)^10^. The present work, therefore, focused only on a semi-quantitative analysis of density distribution of coral colonies.

In total, 1877 coral colonies were recorded from the 23 video sequences, belonging to *Lepidisis* sp., *Anthogorgia* sp., *Calyptrophora* sp., and other forms of the Chrysogorgiidae and Stylasteridae. The abundance of corals varies mostly between 20 and 140 colonies per 100 m^2^. The maximum density occurred at the water depth of 1199 m at the Ganquan slope, reaching 225.6 colonies per 100 m^2^ (Table 1).
